# Transcriptome Analysis and HPLC Profiling of Flavonoid Biosynthesis in *Citrus aurantium* L. during Its Key Developmental Stages

**DOI:** 10.3390/biology11071078

**Published:** 2022-07-19

**Authors:** Jing Chen, Yaliang Shi, Yicheng Zhong, Zhimin Sun, Juan Niu, Yue Wang, Tianxin Chen, Jianhua Chen, Mingbao Luan

**Affiliations:** Institute of Bast Fiber Crops, Chinese Academy of Agricultural Sciences, Key Laboratory of Stem-Fiber Biomass and Engineering Microbiology, Ministry of Agriculture, Changsha 410205, China; cjibfc@sina.com (J.C.); syl_email107254@163.com (Y.S.); zhongyc95@163.com (Y.Z.); szm8634851@163.com (Z.S.); 18530982362@163.com (J.N.); 13378918497@163.com (Y.W.); ctx15974162753@163.com (T.C.)

**Keywords:** *Citrus aurantium* L., transcriptome, flavonoid

## Abstract

**Simple Summary:**

Flavonoid is an important secondary metabolite with rich biological activity and pharmacological activity, and *Citrus aurantium* L. is of value in this regard. *Citrus aurantium* L. fruits are rich in flavonoid, but research information on flavonoid biosynthesis of *Citrus aurantium* L. is rare. Therefore, analysis of the key developmental stage and genes for flavonoid biosynthesis is essential for breeding *Citrus aurantium* L. varieties with high flavonoid content. Here, we report the profile of flavonoid and key flavonoid biosynthesis genes in the growth period of *Citrus aurantium* L. based on transcriptome analysis. We found that total flavonoid content decreased gradually during the fruit development stage, and that neohesperidin was the main flavonoid in the early development stage but with the progression of the development stage, naringin content increased rapidly and became the main flavonoid component. In addition, the key genes related to flavonoid biosynthesis in *Citrus aurantium* L. were identified. These results will lay the foundation for the mechanism underlying flavonoid biosynthesis in *Citrus aurantium* L. fruits.

**Abstract:**

*Citrus aurantium* L. (sour orange) is a significant Chinese medicinal and fruit crop rich in flavonoids. However, the pathways and genes involved in flavonoid biosynthesis at the key developmental stages of *Citrus aurantium* L. are not fully understood. This study found that the total flavonoid concentration gradually decreased as the fruit developed. Additionally, it showed that neohesperidin was the main flavonoid in the early stages of sour orange fruit development. However, as the development stage progressed, naringin content increased rapidly and emerged as the main flavonoid component. From 27 cDNA libraries, RNA sequencing yielded 16.64 billion clean bases, including 8989 differentially expressed genes. We identified 74 flavonoid related unigenes mapped to the phenylalanine, tyrosine, and phenylpropanoid biosynthesis pathways. A total of 152 UDP-glucuronosyltransferase genes (UGTs) were identified from *C. aurantium* L. transcriptome database, in which 22 key flavonoid-correlated UGTs were divided into five main AtGT groups: E, G, I, L, M. We observed that the ethylene responsive factors (ERF) and myeloblastosis (MYB) family mainly regulated the key genes involved in flavonoid biosynthesis. Overall, our study generated extensive and detailed transcriptome data on the development of *C. aurantium* L. and characterized the flavonoid biosynthesis pattern during its fruit developmental stages. These results will benefit genetic modification or selection to increase the flavonoid content in sour oranges.

## 1. Introduction

Sour orange (*Citrus aurantium* L.), a member of the *Rutaceae* family and gene citrus, is said to have its origins in southern China, northern Burma, and northeastern India [1]. Traditionally, sour oranges are usually utilized as a flavoring and acidifying agent for food [2,3]. Due to its substantial medicinal significance, the unripe fruit of sour orange, also known as *Aurantii Fructus*, is regarded as an important economic crop [4,5]. Flavonoids belonging to phenolics have been recognized as important secondary products in the fruits of sour orange [6]. The flavonoids in sour orange have four main groups: flavones, flavanones, flavonols, and anthocyanins [7]. Flavonoids are mainly present in sour orange fruits as glycosyl derivatives including naringin, hesperidin, neohesperidin, narirutin, tangeretin, and poncirin [8]. They are flavonoid compounds with a typical C6–C3–C6 skeleton structure. The naringin and neohesperidin concentrations of sour orange ranged from 1.80 to 26.30 and from 3.90 to 14.71 mg/g, respectively [9]. Flavonoids are the most bioactive plant secondary metabolites and display various physiological functions during plant growth, fruit development, and stress responses. For example, flavonoids in seed coats of *Phaseolus vulgaris*, particularly pelargonidin3-glucoside, inhibit the growth of pathogenic microbes and stimulate the growth of symbiotic bacteria [10]. In addition, flavonoids are involved in the regulation of abiotic and biotic stress responses through REDOX reactions [11,12]. Moreover, flavonoids also exhibit health benefits, including antiulcer [13], anti-inflammatory [14], antitumor [15], antiviral [16], and antioxidant [17] activities. Therefore, it is becoming increasingly important to understand the biosynthesis mechanism of plant flavonoids, and to improve beneficial flavonoid content through breeding.

The flavonoid biosynthesis pathway in plants is a branch of the phenylpropanoid pathway [18]. Chalcone synthase is the first key enzyme in the flavonoid biosynthesis pathway, and it can catalyze the condensation of 3 malonyl CoA and 1 p-coumaroyl CoA to form naringenin chalcone, which is then transformed to naringenin with catalysis by chalcone isomerase [19]. As an elementary precursor, naringenin, can be transformed into various flavonoid classes, including flavones, flavonols, anthocyanins, and proanthocyanidins through various modification reactions under the action of different enzymes [7]. Glycosyltransferases (GTs), acyltransferases (ATs), and *o*-methyltransferase (OMTs) all play a vital role in the structural diversity of flavonoids [20,21,22]. Chen et al. characterized 11 *O*-GTs from *Licorice* and proved that the diversity of *O*-GTs contributed to the biosynthesis of various glycosides in *licorice* [23]. However, studies on the biosynthesis of flavonoids in *C. aurantium* L. have not been reported, and the lack of knowledge of the regulatory mechanisms greatly limits the large-scale development for high flavonoid content.

In this study, three different *C. aurantium* L. accessions, designed as “YJ01”, “YJ33”, and “YJ50”, were chosen as research materials to investigate the mechanisms of transcription during the fruit development stage. Fruits were harvested in eight phases, beginning at 45 DAFB (days after full blooming) and then every 15 days until 150 DAFB.

We then carried out physiological characteristics analysis for 72 samples and found that the total flavonoid content decreased as the growth interval increased. There were significant differences in flavonoid content between the S1, S4, and S7 stages. Finally, three stages (S1, S4, and S7) were selected to conduct a transcriptome analysis of 27 samples.

Our main objectives were to (1) establish the mRNA libraries of *C. aurantium* L.; (2) screen for differently expressed genes related to flavonoids biosynthesis; (3) construct a co-expression network of transcription factors (TFs) correlated with flavonoid biosynthesis and key enzyme-encoding genes in the pathway. The study aims to provide a basis for the understanding of the molecular mechanisms of flavonoid biosynthesis in *C. aurantium* L.

## 2. Materials and Methods

### 2.1. Plant Sample Collection

*C. aurantium* L. was cultivated in the experimental field of Bast Fiber Crops, Chinese Academy of Agricultural Sciences (Yuanjiang, Hunan province). ‘YJ01’, ‘YJ33’, and ‘YJ50’ were selected for study because of the significant difference in flavonoid level (naringin and neohesperidin) between them. *C. aurantium* L. plants are fertilized three times each year in March, August, and November, and were in good condition with healthy and plentiful fruit. Three different *C. aurantium* L. accessions were not far apart and grew in similar conditions. Samples were collected during the fruit development stage from May to September 2019. Fruits were harvested at 45 DAFB (the day after full blooming) and then every 15 days until 150 DAFB. At each stage, four fruits located in the southeast and northwest were mixed as one biological replicate for each sample, and each stage had three replicates. The collected fruits were divided into two parts; one part was quickly put into liquid nitrogen and stored in an ultra-low freezer (−80 °C) for transcriptome sequencing, and the other part was used for flavonoid analysis.

### 2.2. Flavonoid Content Analysis

Fruits harvested at 45, 60, 75, 90, 105, 120, 135, and 150 DAFB were dried to a constant weight at 50 °C and ground to powder. The flavonoids in the powder were extracted by reflux extraction using 90% ethanol as the solvent at 85 °C for 1.5 h and repeated three times. The extract was combined and condensed by vacuum rotary evaporation and resuspended in deionized water. The samples were then analyzed using high-performance liquid chromatography (HPLC, Agilent 1260, USA) with 0.1% acetic acid water and acetonitrile used as the mobile phase at a flow rate of 0.8 mL/min and 30 °C [24]. The flavonoid content in the fruit was determined by an external standard method based on comparing the area of the peak with the known standard (neohesperidin, naringin) concentration.

### 2.3. cDNA Library Construction and Sequence Analysis and Alignment

Total RNA from three accessions developmental stages (45 DAFB, 90 DAFB, and 105 DAFB) were extracted using the RNA prep Pure Plant kit (Tiangen, Beijing, China), and RNA concentration and purity were determined using a Nano Drop Agilent 2100 bioanalyzer (Thermo Fisher Scientific, Waltham, MA, USA). For RNA-sequencing, 27 cDNA libraries were constructed in three stages (each stage had three replicates). mRNA was purified from 1 μg of total RNA, fragmented, and then used to prepare a cDNA library using the NEBNext Ultra RNA Library Prep Kit (Illumina; NEB, Ipswich, MA, USA). cDNA library quality was assessed using the Agilent Bioanalyzer 2100 system (Agilent Technologies, Palo Alto, CA, USA). Illumina sequencing was performed by DNBseq using the Phred +33 quality system. Reads containing poly-N and low-quality reads were removed using SOAPnuke software [25], and the remaining clean reads were aligned to the reference *Citrus sinensis* genome (*Citrus sinensis* v3.0) using HISAT2 (v2.1.0) [26], from which unigenes were obtained.

### 2.4. Differentially Expressed Genes (DEGs)

Mapped reads were counted using feature counts for each sample. Gene expression levels with the FPKM values were estimated using our in-house python script by following the formula: FPKM = total exon fragments/mapped reads (Millions) × exon length (KB) [27,28]. The gene count matrix was used to identify the differentially expressed genes (FDR ≤ 0.05, |fold-change| > 1) by DE-Seq2 packages [29]. The differentially expressed genes were obtained by pairwise comparison between different stages and pairwise comparison between different accessions in the same stage. GO and KEGG enrichment were performed at the OmicShare platform (accessed on 8 March 2022, www.omicshare.com/tools) [30]. Transcription factors were predicted using the Plant Reg Map online database (accessed on 3 Apirl 2022, http://planttfdb.gao-lab.org/prediction.php).

### 2.5. Weighted Correlation Network Analysis (WGCNA) and Coexpression Network Construction

A weighted gene co-expression network was constructed using the Sangerbox tool (accessed on 22 March 2022, https://sangerbox.com/) [31]. Genes differentially expressed in the three accessions were selected for this analysis and 7401 DEGs were used to construct an unsigned gene co-expression network. The soft threshold in this study was 7.0, the min-Module size was 30, and the module merge threshold was 0.25.

The co-expression network image was set up using Cytoscape software [32]. Gene information was obtained from Uniprot database [33].

### 2.6. RT-qPCR Analysis

Quantitative reverse transcription-PCR (RT-qPCR) was employed to validate the RNA-seq data. Total RNA extracted from 27 samples (three accessions, three stages, and three replicates) were reverse-transcribed to cDNA using the PrimeScript RT Master Mix for qPCR (Takara Biotechnology Co., Ltd., Dalian, China). The specific primers for 14 genes related to flavonoid biosynthesis and the internal control primers are shown in Table S6. Relative expression levels were calculated using the 2^−ΔΔCt^ method [34].

## 3. Results

### 3.1. Quantitation of Flavonoid Contents in Citrus aurantium L.

**Figure 1:**
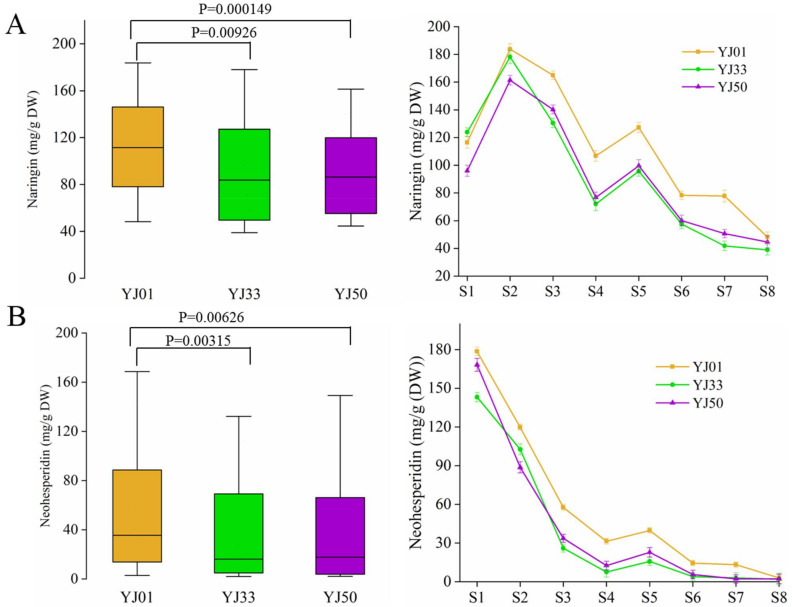
The content of two predominant flavonoids in eight fruit developmental stages of YJ01, YJ33, and YJ50. (**A**) The contents of naringin. (**B**) The contents of neohesperidin. The left boxplot displays the total flavonoid content in YJ01, YJ33, and YJ50. while the right line plot represents the flavonoid level trends in eight fruit developmental stages in YJ01, YJ33, and YJ50. Yellow, green, and purple lines represent YJ01, YJ33, and Y50, respectively. S1/S2/S3/S4/S5/S6/S7/S8 on the *X* axis represent 45/60/75/90/105/120/135/150 days after full bloom; mg/g DW on the *Y* axis represent mg/g dry weighting (DW); “*p*” means *p*-value of the total flavonoids content between YJ01, YJ33, and YJ50.

We determined the total levels of the two flavonoids. Among the two, neohesperidin was found to be a major component in YJ01, YJ33, and YJ50 during S1, and naringin was significantly increased post S1 stage (Table 1). The levels of the two types of flavonoids all behaved with a decreasing trend during all eight stages, and the level of neohesperidin decreased more significantly and earlier than naringin (Figure 1). Specifically, neohesperidin levels in the three accessions decreased to below 50% in the S3 stage (Figure 1B). In contrast, the naringin content remained at a higher level before the S7 stage (Figure 1A). When scanning both flavonoids between these three accessions, we found that naringin and neohesperidin levels were significantly higher in YJ01 than in YJ33, and YJ50 during most developmental stages (Figure 1, Table 1). These results suggest that the major flavonoid in YJ01, YJ33, and YJ50 was naringin during most of the developmental stages and that YJ01 had a much higher level of flavonoid than that of YJ33 and YJ50.

### 3.2. Phylogenetic Analysis and the Expression Landscape between the Three Accessions

**Figure 2:**
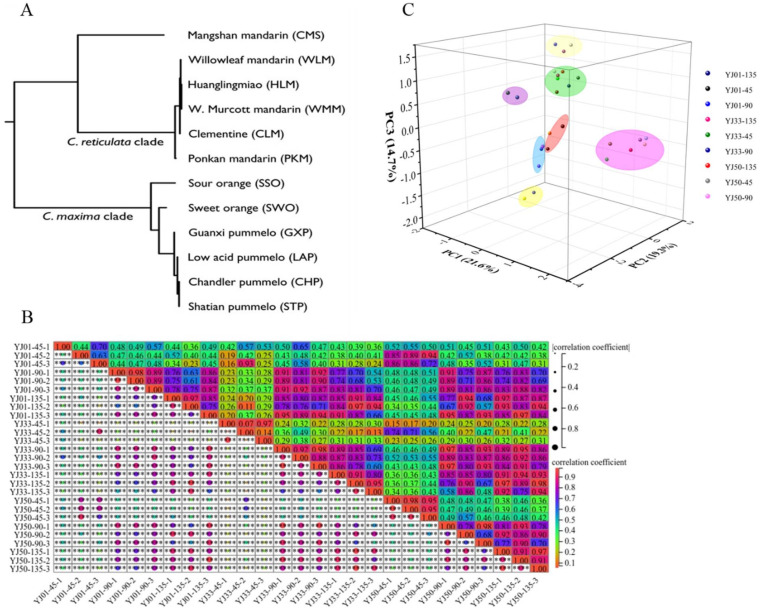
Phylogenetic tree, principal component analysis (PCA), and Pearson correlation co-efficient (PCC) analysis. (**A**) Midpoint-rooted neighbor-joining phylogenetic tree of the *Citrus* chloroplast genomes. The figure was quoted from the reference ‘Sequencing of diverse mandarin, pummelo and orange genomes reveals complex history of admixture during citrus domestication’. (**B**) Pearson correlation co-efficient analysis. FPKM data of 27 samples were used for PCC analysis on the Sangerbox. (**C**) PCA of 27 samples and 45/90/135 represent the days after full blooming.

Edible *Citrus* fruits originated in southeast Asia earlier than several thousand years ago, and spread globally with complex domestication history [35]. Most modern cultivated varieties are typically propagated by grafting and through asexual seed production (apomixis via nucellar polyembryony) to maintain desirable combinations of traits [1,36]. However, the lineages that gave rise to cultivated varieties, have not been recorded without documented antiquity, and the family relationships among *Citrus* fruits remain controversial. Following the published draft genome of many *Citrus* including *Citrus medica*, *Citrus reticulata*, and *Citrus clementina*, some important progress has been made in elucidating the phylogenetic history of *Citrus* domestication. It was reported that the three major ancestors of *Citrus* species including *Citrus reticulata*, *Citrus maxima*, and *Citrus medica* contributed to the origins of all currently cultivated *Citrus* species [37]. Sour orange is a pure F1 hybrid between *C. maxima* (egg donor) and *C. reticulata* (pollen donor) genotypes [38]. Interestingly, sweet orange could be derived only from a cross between (*C. maxima* × *C. reticulata*) × *C. maxima* as an egg donor and a male *C. reticulata*, with some introgression with *C. maxima* [39]. In addition, the midpoint-rooted neighbor-joining phylogenetic tree (Figure 2A) of *Citrus* chloroplast genomes indicated that sweet orange and sour orange had a close relative and phenotypic similarities [40]. When using the *Citrus sinensis* genome as a reference, the average mapping rate of RNA-seq data in YJ01 was 91.92%, in YJ33 92.04%, and in YJ50 91.90% (Supplementary Table S1), indicating that it is satisfactory for further analysis. Therefore, the genome of *Citrus sinensis* was selected as a reference for *Citrus aurantium* L.

Twenty-seven libraries from *C. aurantium* L. fruits collected at three developmental stages (S1, S4, and S7) were sequenced. A total of 16.64 billion clean bases were obtained, with an average of 41,088,594 clean reads and 37,785,209 mapped reads per sample. The average ratio mapped to the reference was 92% (Supplementary Table S1). The Pearson correlation co-efficient (PCC) between any two replicates of the same sample was between 0.68 and 1.0, except for the ‘YJ01-45-2’ and ‘YJ33-45-2’ samples (Figure 2B). The median expression level of all genes in YJ01 gradually declined during the developmental stages, while in YJ33 the expression level increased in the S4 developmental stage, and then decreased in the S7 stage, while the median expression level of all genes in YJ50 was basically stable with a minor change (Supplementary Figure S1). The samples ‘YJ01-45-2’ and ‘YJ33-45-2’ were excluded in subsequent analysis.

Principal component analysis (PCA) of all 27 samples was performed based on RNA-seq FPKM (Figure 2C), and three principal components were found to explain 55.6% of the overall variance (21.6%, 19.3%, and 14.7% for PC1, PC2, and PC3, respectively). Interestingly, it can be observed that in the stage of S1 (45 DAFB), samples from three accessions were clustered together, except for the outlier samples (‘YJ33-45-1’, ‘YJ33-45-3’). These results suggested that the three accessions had highly similar gene expression profiles in the S1 stage (45 DAFB) during fruit development, which might be related to the large amount of secondary metabolites synthesized by fruits in the S1 stage. In addition, the distribution of whole-genome gene expression (FPKMs) values indicated that the S1 stage had a higher total FPKM value than that of the other stages (Supplementary Figure S2).

### 3.3. Identification and Functional Analysis of DEGs during Different Fruit Developmental Stages

**Figure 3:**
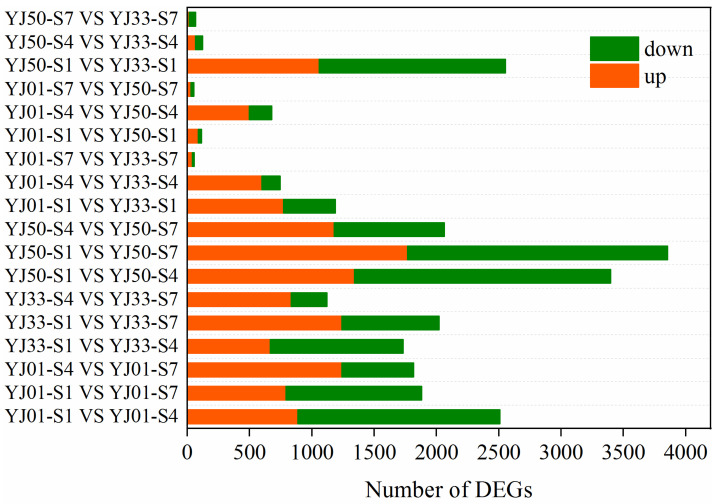
DEGs between YJ01, YJ33, and YJ50 in three fruit developmental stages. Pairwise comparisons of gene expression levels at different stages within each sample, and gene expression levels at the same stage between the two samples.

By pairwise comparison between different stages within each sample, and between two accessions at the same stage, a total of 8989 DEGs were identified according to the criteria of Padj < 0.05, and |log2 fold change| > 1 in each pairwise comparison (Figure 3). The number of DEGs first decreased and then increased as the time interval between the two different stages increased, and the number of DEGs ranged from 14 to 2089 among comparisons. In YJ01 and YJ33, the number of DEGs between S1 and S4 was greater than that between S1 and S7, and this trend was reversed in YJ50. When comparing the two same stages within different accessions, the number of upregulated genes between YJ01 and YJ50 was higher than the number of down-regulated genes, it was identical between YJ01 and YJ33. Among these DEGs, 26 of 47 flavonoid-related biosynthesis genes were differentially expressed in the two different stage comparisons, which accounted for 55% of the total flavonoid-related pathway genes in *C. aurantium* L. (Table 2). Moreover, 12 flavonoid-related DEGs were down-regulated in at least one strain in the two different stage comparisons, while 12 flavonoid-related DEGs showed up-regulation in at least one accession in the two different stages comparisons. Of the 26 flavonoid-related DEGs, nine genes (Cs_ont_7g004690.1, Cs_ont_1g002510.1, Cs_ont_5g038970.1, Cs_ont_6g019320.1, Cs_ont_1g006760.1, Cs_ont_4g024900.1, Cs_ont_1g024230.1, Cs_ont_1g024260.1, and Cs_ont_9g014840.1) were down-regulated in at least two accessions during any two of the stages. These flavonoid-related DEGs were predominately down-regulated during stages, indicating that these genes may contribute to flavonoid accumulation during fruit development. In addition, many genes related to flavonoid biosynthesis in other plants were found in the DEG sets. For example, the phenylalanine ammonia lyase (PAL) gene, a member of the aromatic amino acid lyase family, is strongly associated with increased flavonoid biosynthesis during postharvest senescence in broccoli [41]. We identified five differentially expressed genes (Cs_ont_6g020600.1, Cs_ont_6g020620.1, Cs_ont_8g005310.1, Cs_ont_7g006400.1, and Cs_ont_5g025830.1) of the aromatic amino acid lyase family from *C. aurantium* L. transcriptome database. The expression levels of four DEGs (Cs_ont_6g020600.1, Cs_ont_6g020620.1, Cs_ont_8g005310.1, and Cs_ont_7g006400.1) were subsequently down-regulated in the S1, S4, and S7 stages, which is consistent with the variation of flavonoid content, while the gene (Cs_ont_5g025830.1) exhibited an opposite expression trend. These results suggest that PAL genes might play an important role in the accumulation of flavonoid biosynthesis. Dai et al. identified two UDP-glycosyltransferases (UGTs) genes from *Camellia sinensis*, and elucidated that the two CsUGTs are involved in the biosynthesis of bitter flavonoid 7-O-neohesperidoside through the sequential glucosylation and rhamnosylation of flavonoids [42]. Many studies have reported that UDP-glycoseglycosyl-transferases (UGTs) catalyze the biosynthesis of flavonoid glycosides in plants. A total of 152 UGTs genes were identified from *C. aurantium* L. transcriptome database in our study, and 83 UGT genes displayed different expression levels in the interval stages (Supplementary Table S2). Twenty-seven UGT DEGs were down-regulated during S1, S4, and S7, indicating that these genes may be closely correlated with flavonoid accumulation.

### 3.4. Weighted Gene Co-Expression Network Construction and Analysis

**Figure 4:**
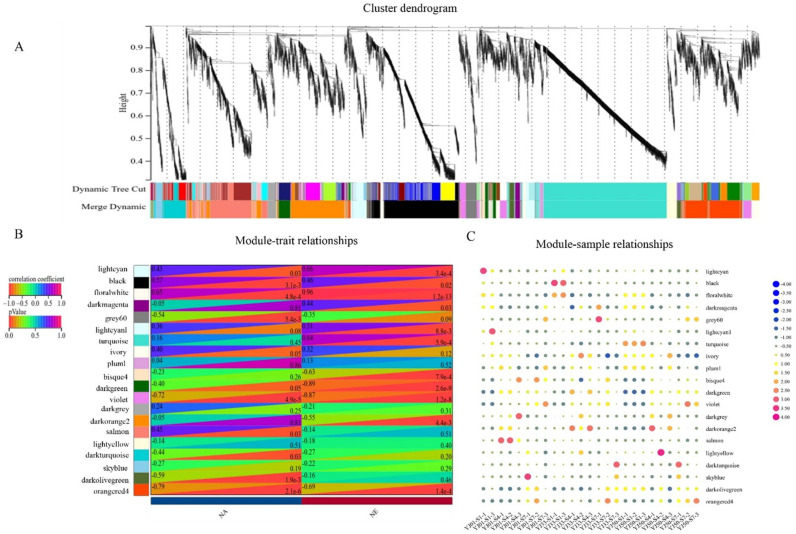
WGCNA network analysis of 25 samples. (**A**) Dendrogram of co-expression modules identified by WGCNA. Genes make up the leaves of the cluster tree, and the below colors represent the annotations of genes. (**B**) Module and flavonoid correlation heatmap. The colorful scale on the left shows correlations from −1.0 to 1.0 and *p*-values from 0 to 1.0. NA and NE represent naringin and neohesperidin, respectively. (**C**) Module-sample correlation heatmap. The colorful scale on the right shows correlations from −1.0 to 1.0. S1, S4, and S7 represent 45, 90, and 135 days after full blooming and 1/2/3 represent three replicates.

To further elucidate which specific genes are highly associated with flavonoid biosynthesis in the fruit development of *C. aurantium* L., the transcriptomic changes were examined by weighted correlation network analysis (WGCNA). DEGs from pairwise comparisons between each time point within the same strain, and between different accessions at one time point were used in a weighted correlation network analysis (WGCNA). The samples: “YJ01-45-2” and “YJ33-45-2” were not considered for WGCNA. In total, 8989 DEGs were used to construct the co-expression network, and 7401 genes were classified into 20 modules (the grey module was excluded) (Figure 4A). Concerning the relationships between modules and physiological traits, we found that the floral-white, light-cyan, and turquoise modules had higher correlations with neohesperidin, and the floral-white and black modules were closely related to naringin content (Figure 4B). We also evaluated the correlation between these modules and gene expression profiles. Highly correlated relationships between gene significance (GS) scores and module membership (MM) in these modules were found. For example, GS and MM had the highest correlation (cor = 0.91, *p* = 8.6 × 10^−49^) in the floral-white for neohesperidin (Supplementary Figure S3). GS and MM also had a higher correlation (cor = 0.53, *p* = 4.6 × 10^−115^) in the turquoise module for neohesperidin (Supplementary Figure S3). GS and MM also had a higher correlation (cor = 0.54, *p* = 7.0 × 10^−11^) in the floral-white module for naringin (Supplementary Figure S3). These results indicated that the genes in these modules were strongly related to flavonoids.

**Figure 5:**
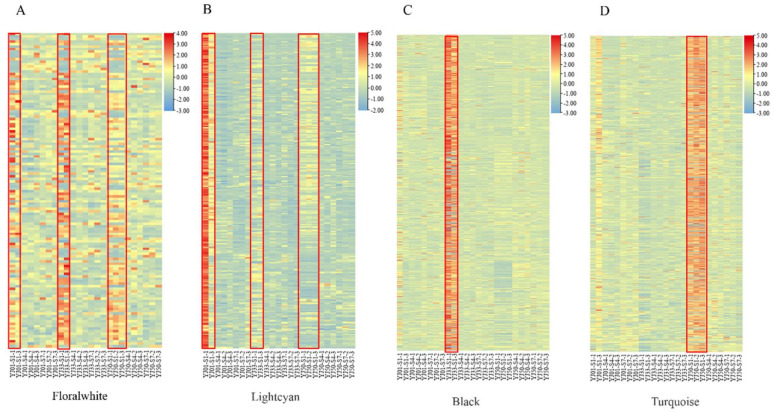
Expression heat maps for all genes in the four key modules. (**A**) The expression profile of 126 genes in the floralwhite module. Samples from YJ01-S1, YJ33-S1, and YJ50-S1 are marked by the red box. S1 represents 45 DAFB and 1/2/3 represents the replicates. (**B**) The expression profile of 264 genes in the lightcyan module. Samples from YJ01-S1, YJ33-S1, and YJ50-S1 are marked by the red box. (**C**) The expression profile of 1119 genes in the black module. Samples from YJ33-S1 are marked by the red box. (**D**) The expression profile of 1601 genes in the turquoise module. Samples from YJ50-S1 are marked by the red box.

**Figure 6:**
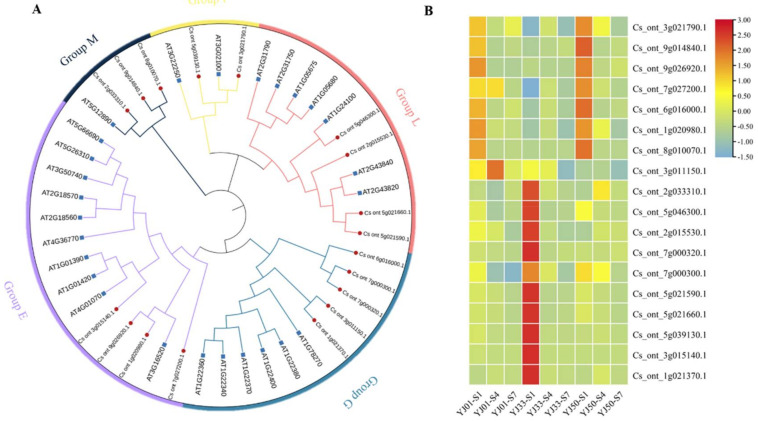
Glycosyltransferase (GT) unigenes identified in *C. aurantium* L. (**A**) phylogenetic analysis of *AtGTs* (*Arabidopsis thaliana*), and *CaGTs* (*Citrus aurantium* L.) using protein sequences. (**B**) Heat map of *CaGTs* based on FPKM.

By analyzing the module-sample correlation heat map, we also found that the floral-white, light-cyan, turquoise, and black modules were stage-specific or sample-specific (Figure 4C). The floral-white and light-cyan modules were specifically correlated during the S1 stage in YJ01, YJ33, and YJ50. The turquoise module was correlated with YJ50 during the S1 stage. The black module was correlated with YJ33 during the S1 stages. In 25 samples, genes in the floral-white and light-cyan modules were highly expressed at 45 DAFB in all tested accessions; genes in the turquoise module were more highly expressed in YJ50 than those in YJ33 and YJ01 during S1; genes in the black module were more highly expressed in YJ33 than those in YJ01 and YJ50 during S1. The gene expression profiles of these modules were consistent with the module-sample correlation heat map (Figure 5A–D). Genes in the floral-white and light-cyan modules were expressed more highly in S1 than in any other stage, which may have led to the higher flavonoid content in S1 than in any of the other stages (Figure 5A,B). We identified 40 UGT DEGs in the four key modules, and 22 UGT genes were repeated with 27 UGTs screened in the transcriptome (Supplementary Table S4). These results suggested that these 22 UGT genes were conducive to the biosynthesis of flavonoids during the developmental stages of sour orange. Using the neighbor-joining (NJ) method in the MEGA X software, these key UGT candidate protein sequences were constructed together with *Arabidopsis* UGT protein sequences to build a phylogenetic tree (Figure 6A). This indicated that these candidate UGTs had similar protein sequences to those in *Arabidopsis* and they all had the GT domain and the conservative PSPG-box motif [43,44]. There are four main UGT subfamilies in *C. aurantium* L.: Groups E, G, I, L, and M [45,46]. Six UGTs (Cs_ont_7g000300.1, Cs_ont_7g000320.1, Cs_ont_9g026920.1, Cs_ont_3g021790.1, Cs_ont_9g014840.1, and Cs_ont_7g027200.1) showed high expression levels (FPKM > 100) (Figure 6B).

**Figure 7:**
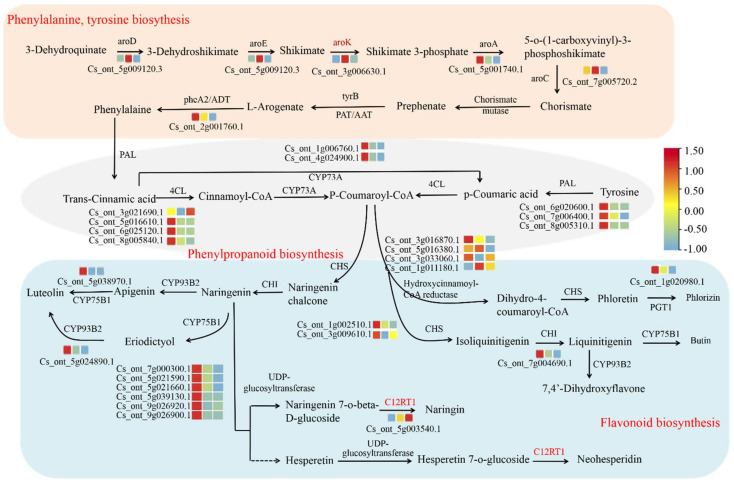
Schematic illustrations of pathways related to flavonoid biosynthesis including the phenylalanine, phenylpropanoid, and flavonoid pathways. Expression profiles of DEGs related to the phenylalanine, phenylpropanoid, and flavonoid pathways. The color scale from red to blue corresponds to expression levels from high to low based on the FPKM. aroD: 3-dehydroguinate dehydratase; aroE: shikimate dehydrogenase; aroK: shikimate kinase; aroA: 3-phosphoshikimate-1-carboxyvinyltransferase; aroC: chorismate synthase; tyrB: aromatic-amino-acid transaminase; PAT/AAT: bifunctional; pheA2: prephenate dehydratase; ADT/PDT: arogenate/prephenate dehydratase; PAL: phenylalanine ammonia lyase; 4CL: 4-coumarate—CoA ligase; CYP73A: trans-cinnamate 4-monooxygenase; CHS: chalcone synthase; CHI: chalcone isomerase; C12RT1: flavanone 7-O-glucoside 2″-O-beta-L-rhamnosyltransferase; PGT1: phlorizin synthase; CYP75B1: flavonoid 3’-monooxygenase; CYP93B2: flavone synthase II. Names of genes in red means DEGs except for the four key modules identified from WGCNA.

Furthermore, we analyzed the key genes related to flavonoid biosynthesis in the four key modules and constructed a model of flavonoid biosynthesis in sour orange fruit, as shown in Figure 7. A total of 74 unigenes related to flavonoid biosynthesis were identified, including five unigenes for phenylalanine, and tyrosine biosynthesis, nine unigenes for phenylpropanoid biosynthesis, and 60 unigenes for flavonoid biosynthesis. Among the 74 unigenes, 64 belonged to the four key modules (turquoise, black, light-cyan, floral-white) (Supplementary Table S4). Most genes involved in flavonoid synthesis showed higher expression levels at 45 DAFB (Figure 7). Thus, four main modules were identified. These modules were closely associated with flavonoid biosynthesis, and many flavonoid-related DEGs were identified in these modules, which were selected as candidate genes for further investigation.

### 3.5. Identification of Transcriptional Regulator Networks

**Figure 8:**
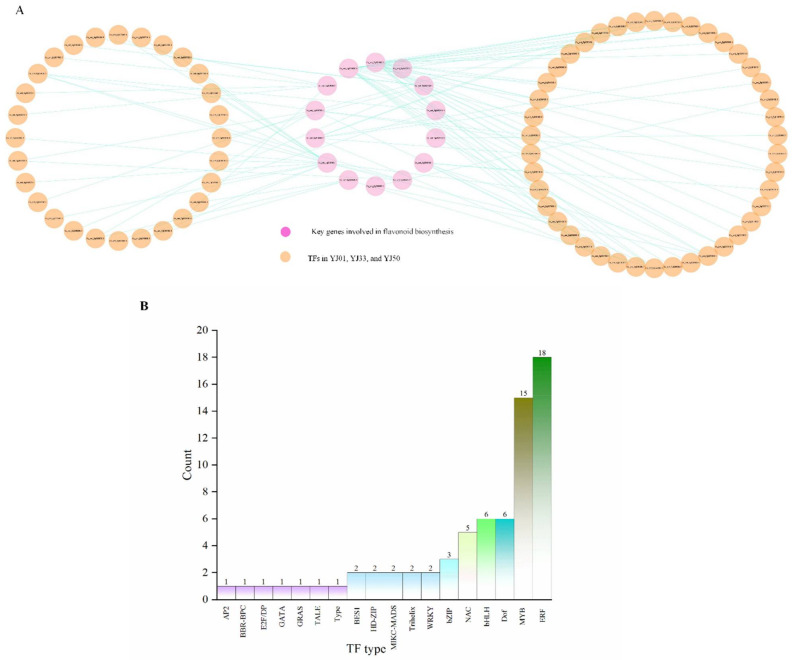
Regulatory network of YJ01, YJ33, and YJ50 and the statistics of TFs in their co-expression network. (**A**) Red circle represents the key genes involved in flavonoid. Orange circle represents TFs predicted in YJ01, YJ33, YJ50. (**B**) The number of different TF-types in the network.

Based on WGCNA and DEG analysis, 14 genes were selected from the flavonoid-related DEGs to predict the main transcription factors involved in regulating the key flavonoid-related structural genes. These genes were in the four key modules exhibiting a high correlation with flavonoid accumulation; therefore, they were presumed to be the key genes involved in flavonoid biosynthesis. These genes included CHI (Cs_ont_7g004690.1), CYP75B1 (Cs_ont_5g038970.1), CYP73A (Cs_ont_4g024900.1, Cs_ont_1g006760.1), PGT1 (Cs_ont_1g020980.1), CYP93B2 (Cs_ont_5g024890.1), UDP-glycosyltransferase (Cs_ont_9g014840.1, Cs_ont_8g010070.1), CHS (Cs_ont_3g009610.1, Cs_ont_1g002510.1), 4CL (Cs_ont_6g025120.1, Cs_ont_8g005840.1), and PAL (Cs_ont_6g020600.1, Cs_ont_8g005310.1). A total of 101 transcription factors (TFs) were predicted, and one transcriptional regulator network (TRN) was constructed with these 14 genes (Supplementary Table S5). Finally, 70 TFs were co-expressed in *C. aurantium* L. fruit development (YJ01, YJ33, and YJ50), suggesting the three *C. aurantium* L. accessions have the same regulatory networks (Figure 8A). In the transcriptional regulator network, we identified 70 TFs that were co-expressed with 14 key genes. These TFs were mostly from the ERF family (eighteen), MYB family (fifteen), Dof family (seven), bHLH family (six), and NAC family (five) (Figure 8B).

### 3.6. Validation of RNA-Seq

**Figure 9:**
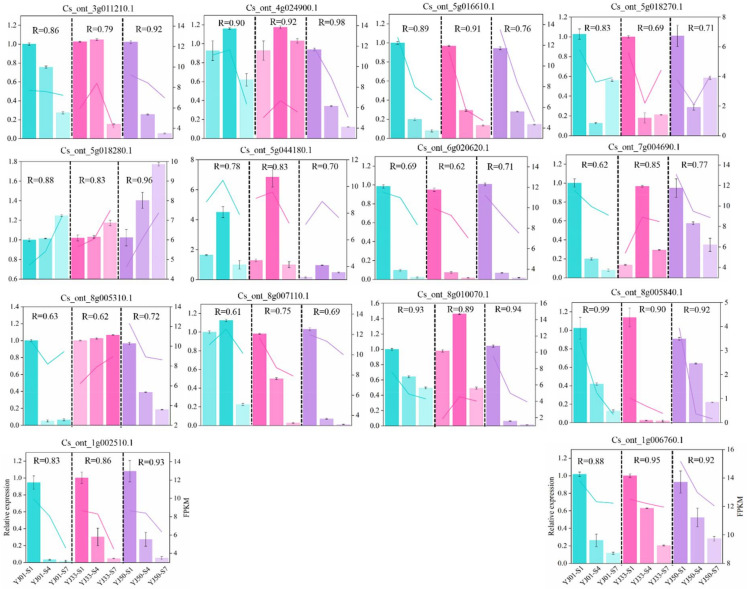
Validation of RNA-seq expression profiles by RT-qPCR. The left *Y*-axis represents relative expression levels corresponding to the bar plot with standard deviation (SD, marked as the error bar in each plot). The right *Y*-axis represents gene expression levels calculated by the fragments per kilobase per million reads (FPKM) method, and FPKM is denoted as the line plot in each plot. The *X*-axis means three fruit developmental stages in YJ01, YJ33, and YJ50. S1/S4/S7: 45/90/135 days after full blooming. R means Pearson correlation coefficient (PCC) between relative expression and FPKM.

To validate the accuracy of the RNA-seq data, we selected 14 DEGs at random for analysis of their expression levels by RT-qPCR, and specific primers were designed for these genes (Supplementary Table S6). The Pearson correlation coefficient (PCC) was calculated to assess the correlation between the relative expression values and FPKM. Nine genes presented PCC values higher than 0.7 in all the samples. The gene expression trend exhibited high consistency between the RNA-seq and RT-qPCR results (Figure 9). We conclude that our transcriptome data can be used for further analysis.

## 4. Discussion

Flavonoids, a class of major secondary metabolites in plants, not only play significant roles in regulating the physiological function of the plant, but also benefit human health with essential pharmacological activities. Understanding the regulatory mechanisms of flavonoid synthesis and identifying key genes to improve the content of flavonoid compounds have been extensively investigated in *Citrus* plants [47,48,49], while few research studies have paid attention to *C. aurantium* L. Given that *C. aurantium* L. is a significant economic fruit tree and flavonoids are one of the most important bioactive compounds, it is important to identify and elucidate the function of genes involved in flavonoid biosynthesis comprehensively and systematically at the transcriptional level. In the present study, we mainly focused on the key structural enzymes and transcription factors involved in the biosynthesis of flavonoid scaffold molecules using comparative transcriptome analysis. The results are conducive to our knowledge of the flavonoid control network in *C. aurantium* L., the accumulation of flavonoids during fruit developmental stages, and the associated molecular mechanisms, which lay the foundation for research on flavonoid synthesis.

The flavonoid biosynthesis pathway is a branch of the phenylalanine metabolic pathway. Coumaroyl coA is formed from phenylalanine under the catalysis of phenylalanine ammonia-lyase, cinnamate-4-dehydrogenase, and 4-coumarate-CoA ligase, and it enters the flavonoid–anthocyanin pathway under mediation by CHS [50]. Further reactions show that chalcone is isomerized by CHI to produce naringenin. Naringenin, the common substrate of all flavonoids, is converted to various kinds of flavonoids through catalysis and derivation of enzymes from downstream pathways [51]. In the present study, 74 DEGs involved in flavonoid synthesis were identified among the YJ01, YJ33, and YJ50 groups, including PAL, 4CL, CHI, CHS, CYP75B1, CYP73A, PGT1, CYP93B2, and UDP-glycosyltransferase. The listed key structural genes correlating with flavonoid synthesis in sour oranges are flavonoid-related genes reported in other plants. Flavonoid glycoside derivatives are a common form of flavonoid produced in plants. In this study, naringin and neohesperidin accounted for the highest proportion of the total flavonoids present in *C. aurantium* L. Many studies have reported that UDP-glycoseglycosyltransferases (UGTs) catalyze the biosynthesis of flavonoid glycosides in plants. In *Citrus*, it has been reported that naringenin-specific 7-O-glycosyltransferase could catalyze the glucosylated naringenin to produce naringenin 7-O-glucoside, and the latter transfers to bitter 7-O-neohesperidoside (naringenin-neohesperidoside) under 1-2-rhamnosyltransferase or to tasteless 7-O-rutinoside (naringenin 7-O-rutinoside) under 1-6-rhamnosyltransferase [52]. However, UGT genes involved in the synthesizing of flavonoid glycosides in *C. aurantium* L. plants have not been cloned and functionally characterized. Identification of the key genes involved in the biosynthesis of flavonoid glycosides in *C. aurantium* L. is important to understand the mechanism of flavonoid synthesis. We obtained 152 UGT family genes through gene family analysis and gene annotation based on *C. aurantium* L. transcription data and 83 UGT family DEGs were analyzed by differential expression analysis, and 22 key UGTs related with flavonoids were screened through WGCNA and gene expression pattern, suggesting that these 22 UGT genes might play a crucial role in flavonoid glycosides and deserve further analysis.

The biosynthesis of flavonoids in plants is regulated by both structural function genes and transcription factors. TFs exert positive or negative regulation on the expression levels of structural function genes by binding to recognition sites in target gene promoters. It has been reported that TFs of the MYB, bHLH, Dof, and ERF protein families are involved in regulating flavonoid biosynthesis [47,53,54]. MYB, one of the biggest transcription factor families, has been identified in many plants, for example, csMYB2 and csMYB26 in tea plants [55], and PpMYB10.1, PpMYB10.2, and PpMYB9 in peach [56]. MYB TFs generally regulate structural genes encoding enzymes involved in the early stages of the flavonoid biosynthesis pathway [57]. However, bHLH factors might play an auxiliary role in the synthesis of anthocyanin from the flavonoid metabolism pathway to the anthocyanin biosynthesis pathway [58]. Furthermore, studies have shown that different TF genes and their families, like MYB, bHLH, WD40, and other gene families often form MBW complexes to fine-regulate the biosynthetic pathway of flavonoids [53,59]. Carmen Arlotta et al. reported that the fruit pomegranate polyphenolic composition varies according to cultivar, tissue, and fruit development stage which is probably regulated to a combination of MYB and bHLH type transcription factors [58]. In this study, we conducted a prediction of transcription factors (TFs) of the key genes involved in flavonoid biosynthesis of *C. aurantium* L. fruit development and explored their regulatory co-expression relationships. We found that 14 key flavonoid-related DEGs were mostly regulated by ERF and MYB families. In addition to TFs, such as Dof, bHLH, bZIP, WRKY, and NAC proteins, many other types of TFs are also involved in the regulation of flavonoids, which requires further study.

## 5. Conclusions

Sour orange is a widely cultivated woody fruit tree species and its fruit is rich in flavonoids. However, the critical genes involved in flavonoid biosynthesis in developing sour orange fruits remain unclear. In this study, the different developmental stages of three *C. aurantium* L. accessions with different flavonoid contents were examined. Transcriptome sequencing was conducted to analyze the differentially expressed genes related to flavonoid accumulation and the relationships between gene expression and flavonoid content to develop flavonoid-rich *C. aurantium* L. In the present study, 74 DEGs involved in flavonoid synthesis were identified between the YJ01, YJ33, and YJ50 groups, including PAL, 4CL, CHI, CHS, CYP75B1, CYP73A, PGT1, CYP93B2, and UDP-glycosyltransferase. Twenty-two key UGTs related to flavonoids were screened using WGCNA and gene expression patterns, suggesting that these 22 UGT genes might play a crucial role in flavonoid glycosides and deserve further analysis. Fourteen key flavonoid-related DEGs were selected, and the regulatory networks of these candidate genes were predicted. We identified 14 key DEGs in the flavonoid biosynthesis pathway that were mostly regulated by the ERF and MYB families. Collectively, our study characterized the flavonoid biosynthesis pattern during fruit development and provided large-scale and comprehensive transcriptome data for *C. aurantium* L. fruit development. We also identified the candidate genes involved in the manipulation of flavonoids in sour orange. These results may benefit genetic modification or selection for further improvement in the flavonoid content of sour oranges.

## Figures and Tables

**Table 1:** Digital descriptions of flavonoid contents. Digital descriptions of flavonoid content in Figure 1 (average ± SD).

Flavonoid Type	Varieties	S1	S2	S3	S4	S5	S6	S7	S8
Naringin	YJ01	116.432 ± 1.08	183.78533 ± 0.92	164.96033 ± 0.08	106.728 ± 0.9	127.26367 ± 0.65	78.34 ± 0.11	77.739 ± 1.35	48.221 ± 0.58
	YJ33	123.87 ± 0.12	178.125 ± 1.81	130.525 ± 0.36	71.98 ± 2.9	95.73 ± 0.68	57.575 ± 0.37	41.81 ± 0.48	38.89 ± 0.69
	YJ50	95.9926 ± 1.09	161.3924 ± 0.44	140.2121 ± 0.23	76.74742 ± 0.86	99.62167 ± 1.43	60.0242 ± 0.91	50.6822 ± 0.08	44.6606 ± 1.33
Neohesperidin	YJ01	178.6218 ± 1.31	119.7779 ± 0.24	57.6393 ± 0.4	31.41540 ± 0.25	39.7634 ± 0.14	14.4204 ± 0.15	13.2093 ± 0.12	2.9119 ± 0.04
	YJ33	143.17175 ± 0.53	102.65441 ± 1.21	26.00266 ± 0.08	7.57086 ± 1.04	15.70094 ± 0.25	3.97631 ± 0.05	2.92 ± 1.05	2.009 ± 1.55
	YJ50	168.1867 ± 1.97	88.6930 ± 1.32	33.6265 ± 0.08	12.5768 ± 0.34	22.8530 ± 0.72	5.6430 ± 0.28	2.2437 ± 0.21	2.20816 ± 0.67

**Table 2:** Details of flavonoid-related DEGs. S1, S4, S7 indicate the three stages that occur from 45 to 135 DAFB. (+) means gene up-regulated expression and (−) means gene down-regulated expression.

Genes	Function	YJ01			YJ33			YJ50		
		S1 vs. S4	S1 vs. S7	S4 vs. S7	S1 vs. S4	S1 vs. S7	S4 vs. S7	S1 vs. S4	S1 vs. S7	S4 vs. S7
Cs_ont_3g008580.1	CHS					+	+			+
Cs_ont_7g004690.1	CHI		−					−	−	
Cs_ont_7g019870.1	LAR		+							
Cs_ont_5g040910.1	ANS		+	+	+	+		+	+	+
Cs_ont_3g009610.1	CHS								+	+
Cs_ont_6g025170.1	HHT1		+	+	+	+		+	+	+
Cs_ont_2g006480.1	ANR							−		+
Cs_ont_6g005600.1	SAT									+
Cs_ont_1g002510.1	CHS		−	−		−	−		−	−
Cs_ont_1g014200.1	N/A				+	+	+	+	+	+
Cs_ont_5g038970.1	CYP75B1	−	−					−	−	
Cs_ont_5g024640.1	HST	+	+		+	+	−	+	+	
Cs_ont_8g011010.1	SAT				+				−	
Cs_ont_6g019320.1	N/A		−					−	−	
Cs_ont_1g006760.1	CYP73A	−	−					−	−	
Cs_ont_1g014190.1	CCOMT								+	+
Cs_ont_1g020980.1	PGT1							−	−	−
Cs_ont_5g024870.1	CYP93B16		+			+				
Cs_ont_4g024900.1	CYP73A		−	−				−	−	−
Cs_ont_9g012610.1	CHS1					+				+
Cs_ont_8g017240.5	CHS					+				
Cs_ont_1g002480.1	ACS2		−							
Cs_ont_5g024890.1	CYP93B2							−	−	
Cs_ont_1g024230.1	CYP81Q32			−		−	−	+	+	−
Cs_ont_1g024260.1	CYP81Q32	−	−						−	−
Cs_ont_9g014840.1	UDPGT	−						−	−	

## Data Availability

The materials of this study were provided by the Institute of Bast Fiber Crops, Chinese Academy of Agricultural Sciences. Correspondence and requests for materials should be addressed to Jianhua Chen (chenjianhua@caas.cn). The sequencing data have been deposited in NCBI SRA database (accession number: PRJNA859938).

## Supplementary Materials

The following are available online at https://www.mdpi.com/article/10.3390/biology11071078/s1, Figure S1: Boxplot for FPKM values. 45/90/135 means the days after full blooming. 1/2/3 represent three replicates. Figure S2: Kernel plot of the overall expression density. (A) YJ50 (B) YJ33 (C) YJ01. X axis represent log10(FPKM). Figure S3: The correlations between modules and the gene expression profiles. Table S1: Data description of RNA-seq reads for all samples with three replicates. Table S2: UDP-glycose: glycosyltransferases DEGs identified from *C. aurantium* L. Table S3: Genes in floralwhite, black, lightcyan, and turquise modules. Table S4: key differently expressed genes in flavonoid biosynthesis pattern from floralwhite, black, lightcyan, and turquise modules. Table S5: Identification of transcription factors in co-expression networks. Table S6: specific primers of genes selected for qRT-PCR.
