# Supplementary material for: Transcriptome Analysis and HPLC Profiling of Flavonoid Biosynthesis in Citrus aurantium L. during Its Key Developmental Stages
Source: Biology (Basel). 2022 Jul 19;11(7):1078. doi: 10.3390/biology11071078 (PMC9313048; doi:10.3390/biology11071078)
Supplement: Supplementary file 1 [file biology-11-01078-s001.zip › supplementary figure.pdf]

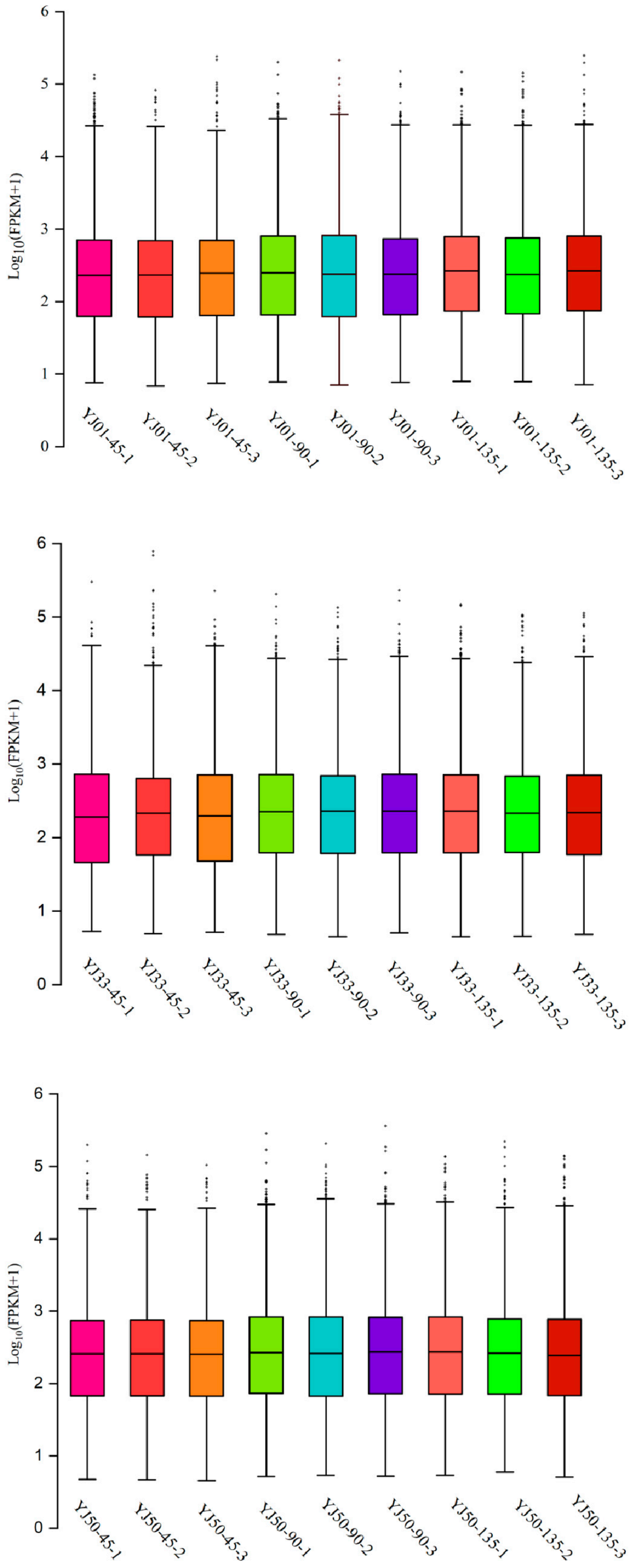

Figure S1. Boxplot for FPKM values. 45/90/135 means the days after full blooming. 1/2/3 represent three replicates.

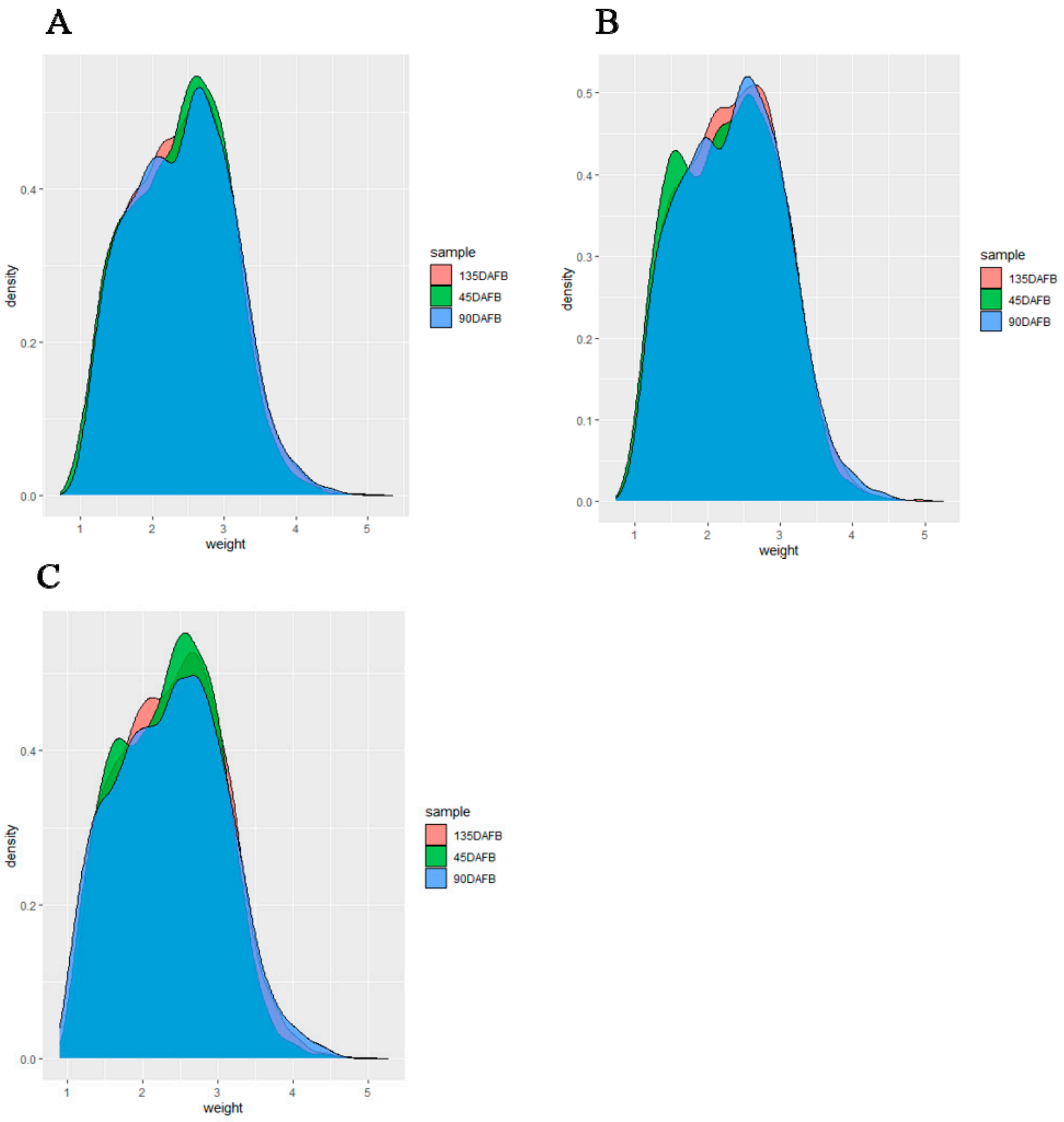

Figure S2. Kernel plot of the overall expression density. (A) YJ50 (B) YJ33 (C) YJ01. X axis represent log10(FPKM).

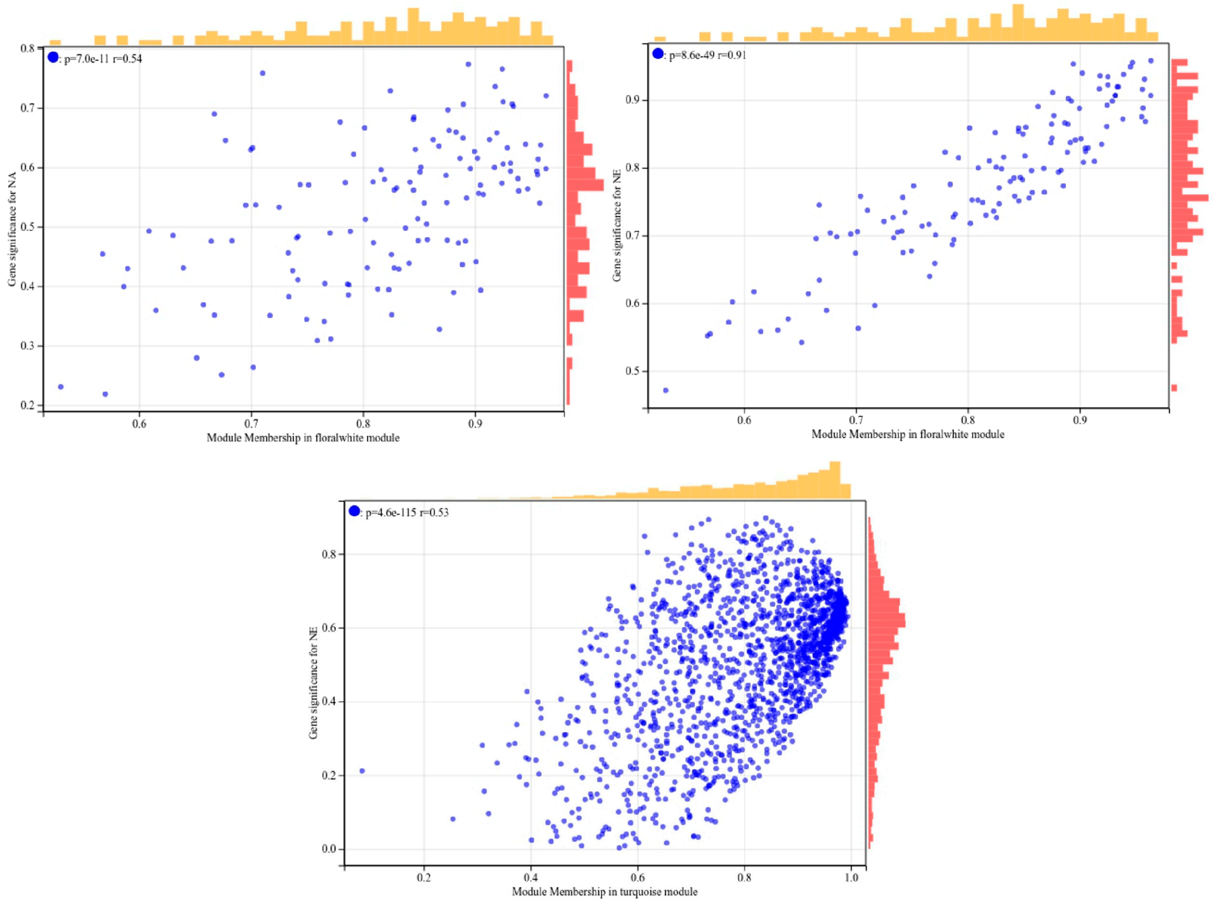

Figure S3. The correlations between modules and the gene expression profiles.
